# Drugs in focus: Budesonide and its role in paediatric gastrointestinal disorders

**DOI:** 10.1002/jpn3.70245

**Published:** 2025-10-27

**Authors:** Sohail Aziz, Maria Giovanna Puoti, Amit Assa, Zrinjka Misak, Emmanuel Mas, Osvaldo Borrelli, Jernej Dolinšek, Javier Martin‐de‐Carpi, Isabelle Scheers, Christos Tzivinikos, Matjaž Homan, Caterina Strisciuglio

**Affiliations:** ^1^ Department of Experimental Medicine University of Campania “Luigi Vanvitelli” Napels Italy; ^2^ Department of Paediatric Gastroenterology and Hepatology Santobono‐Pausilipon Hospital Naples Italy; ^3^ The Pediatric Gastroenterology Unit, Kaplan Medical Center Rehovot and the Hebrew University Jerusalem Israel; ^4^ Referral Centre for Paediatric Gastroenterology and Nutrition, Children's Hospital Zagreb, Medical School University of Zagreb Zagreb Croatia; ^5^ Service de Gastroentérologie, Hépatologie, Nutrition et Maladies Héréditaires du Métabolisme, Hôpital des Enfants, and IRSD Université de Toulouse, INSERM, INRAE, ENVT, UPS Toulouse France; ^6^ Department of Paediatric Gastroenterology, Division of Neurogastroenterology & Motility Great Ormond Street Hospital London UK; ^7^ Department of Pediatrics, University Medical Centre Maribor, and Department of Pediatrics, Medical Faculty University of Maribor Maribor Slovenia; ^8^ Department of Paediatric Gastroenterology, Hepatology and Nutrition Hospital Sant Joan de Déu Barcelona Spain; ^9^ Pediatric Gastroenterology and Hepatology, Department of Pediatrics Cliniques universitaires Saint‐Luc, Université catholique de Louvain Brussels Belgium; ^10^ Paediatric Gastroenterology Department, Al Jalila Children's Specialty Hospital Mohammed Bin Rashid University of Medicine and Health Sciences Dubai UAE; ^11^ Department of Gastroenterology, Hepatology and Nutrition University Children's Hospital, Faculty of Medicine, University of Ljubljana Ljubljana Slovenia; ^12^ Department of Woman, Child and General and Specialist Surgery University of Campania “Luigi Vanvitelli” Naples Italy

**Keywords:** adrenal suppression, children, corticosteroids, eosinophilic esophagitis, inflammatory bowel disease

## Abstract

Budesonide is a glucocorticoid with strong topical anti‐inflammatory properties and minimal systemic effects due to extensive first‐pass hepatic metabolism. It is designed for targeted delivery within the gastrointestinal (GI) tract and is available in oral and rectal formulations. Budesonide is indicated for various GI disorders, including Crohn's disease (CD, ulcerative colitis (UC) and eosinophilic oesophagitis (EoE), with specific formulations approved for different disease locations and severities. This narrative review evaluates the pharmacological profile, clinical applications and guideline recommendations surrounding the use of budesonide in both paediatric and adult GI disorders. Evidence from randomised controlled trials and real‐world studies supports the efficacy of budesonide in inducing remission in mild to moderate ileocaecal CD. It is recommended by ESPGHAN/ECCO [European Society for Pediatric Gastroenterology, Hepatology, and Nutrition/European Crohn's and Colitis Organisation] guidelines as a therapeutic alternative in selected paediatric patients when exclusive enteral nutrition is not feasible. In UC, budesonide‐multimatrix tablets and rectal foam formulations may be effective for distal and left‐sided disease, though they are generally less effective than systemic corticosteroids or 5‐aminosalicylic acid and are reserved for patients with contraindications to standard therapies. In EoE, topical swallowed budesonide has shown promising results in inducing clinical and histological remission, with increasing data supporting its use in children. Although budesonide is associated with fewer systemic adverse effects, long‐term use may still pose risks such as growth suppression and hypothalamic‐pituitary adrenal axis suppression, necessitating careful monitoring. Budesonide offers a valuable treatment option when used in alignment with disease phenotype, formulation properties and patient‐specific considerations.

## Abstract (highlights)

**What is Known:**

- Budesonide is a glucocorticoid with strong local anti‐inflammatory effects and high first‐pass metabolism, minimising systemic exposure.
- It is effective in eosinophilic esophagitis, Crohn's disease, ulcerative colitis and microscopic colitis.
- Compared to systemic corticosteroids, it has fewer systemic side effects but still requires monitoring for hypothalamic‐pituitary adrenal axis suppression.

**What is New:**

- Oral viscous budesonide improves both symptoms and mucosal changes in eosinophilic esophagitis.
- It is now first‐line therapy for microscopic colitis, even in prolonged use.
- Emerging evidence suggests a need for optimising long‐term treatment strategies to balance efficacy with safety, particularly regarding systemic risks.
- Novel delivery systems such as orodispersible tablets and modified‐release suspensions are being developed to enhance local efficacy and reduce systemic exposure.

## 1 INTRODUCTION

Budesonide is a potent corticosteroid with low systemic bioavailability and strong local anti‐inflammatory activity. In the early 1990s, an oral controlled‐release formulation was developed for Crohn's disease (CD), targeting the distal ileum and ascending colon.^1^ To enable delivery to the distal ileum, proximal colon, or entire colon while avoiding gastric degradation, several controlled‐release formulations have been designed. These provide high local drug concentrations while minimising proximal small bowel absorption, thereby reducing systemic exposure and improving efficacy.^2^ Currently, three oral formulations are available: a pH‐dependent‐release version, a multimatrix (MMX) system and a controlled ileal‐release form combining pH‐ and time‐dependent mechanisms. Budesonide is also available as enemas and rectal foams for topical delivery.^3^

Despite its established use, the effectiveness and safety of budesonide particularly its impact on the adrenal axis in infants and children remain underexplored. A clear understanding of its pharmacological profile, indications and safety is essential for optimal paediatric use.

This article aims to provide a summary of the available evidence on the use of budesonide for treating gastrointestinal (GI) disorders in children.

## 2 METHODS

A comprehensive literature search was performed in PubMed, Google Scholar, Scopus and Cochrane Library from January 1990 to May 2024, focusing on paediatric and adult studies evaluating budesonide in gastrointestinal disorders. The following search terms were used in various combinations: ‘budesonide’, ‘Crohn's disease’, ‘ulcerative colitis’, ‘microscopic colitis’, ‘eosinophilic esophagitis’, ‘paediatrics’, ‘children’ and ‘inflammatory bowel disease’. Only English‐language studies were included. Both randomised controlled trials (RCTs) and relevant observational studies (prospective or retrospective cohorts, registry data and case series with ≥10 patients) were considered. Study quality was not formally assessed with scoring tools, given the narrative design, but priority was given to RCTs, large cohort studies and guideline documents. The studies meeting our inclusion criteria have been summarised in Tables 1 and 2, which provide an overview of the evidence base across the major disease categories.

**Table 1:** Budesonide in EoE paediatric patients.

References	Study type	Description and study numbers	Main finding
[4]	Retrospective analysis	20 children (mean age 5.5 years) with EoE treated with oral viscous budesonide.	Significant improvement in eos/hpf (87–7, *p* < 0.0001), symptom score (4.4–0.8), and endoscopy score (3.6–0.8). No significant adverse events.
[5]	Randomised, double‐blind, placebo‐controlled	15 children on budesonide and 9 on placebo, followed over 3 months with biopsies and endoscopie	Peak eosinophil count improved from 66.7 to 4.8 eos/hpf in budesonide group (*p* < 0.01); symptom, endoscopic and histological scores significantly improved. Oral candidiasis observed in some patients.
[6]	Induction and maintenance therapy study	20 paediatric patients (median age 10 years), 12‐week induction +12‐week maintenance +12‐week follow‐up.	90% achieved remission post‐induction (*p* < 0.01); histological, endoscopic and clinical scores improved. Some relapse occurred by Week 36.
[7]	Dose‐ranging, placebo‐controlled	71 patients aged 2–18 years; received placebo or low, medium, or high dose budesonide suspension for 12 weeks.	Significant histologic response (≤16 eos/hpf) with medium and high doses. Symptom response not significantly different. No major adverse events reported.
[8]	Clinical trial	EoE patients with oesophageal atresia treated with oral viscous budesonide for 12 weeks.	87.5% achieved histologic remission; clinical and endoscopic improvements noted. No treatment‐associated adverse events.
[9, 10]	Pilot studies	Short‐term budesonide therapy (8–12 weeks) in paediatric EoE patients.	Demonstrated histological remission; consistent with findings from other studies.

Abbreviations: EoE, eosinophilic oesophagitis; eos/hpf, eosinophil counts per high‐power field.

**Table 2:** Budesonide in IBD.

Reference	Study type	Description and study numbers	Main finding
[11]	Randomised, placebo‐controlled	CD patients treated with 9 mg/day versus 12 mg/day budesonide.	Remission: 42.9% (9 mg) versus 65.7% (12 mg); CRP reduction; no significant adverse events.
[12]	Multicentre clinical trial	108 paediatric CD patients (6–17 years); 9 mg or 6 mg budesonide for 8 weeks.	58.1% remission (*p* < 0.001); reduction in disease activity; 32 patients had subnormal cortisol; good tolerability overall.
[13]	Prospective registry analysis	Newly diagnosed CD patients in North America; 13% received budesonide; 47% within 30 days.	Budesonide monotherapy uncommon (5%); often used with adjunct therapies.
[14]	Prospective analysis	9 paediatric UC patients (mean age 7.5 years); budesonide MMX for 8 weeks.	Clinical remission in 77.7% at 4 weeks; continued efficacy up to 8 weeks; well tolerated.
[15]	Prospective analysis	31 paediatric UC patients (mean age 13.2 years); budesonide MMX for 2 months (3 patients >10 months).	Clinical remission in 55%; endoscopic improvement in 73%; remission in 40%; adverse events in long‐term use.
[16]	Prospective study	16 paediatric UC patients (median age 14.54 years); budesonide MMX for 8 weeks.	Clinical remission in only one patient; high safety but limited efficacy observed.

Abbreviations: CD, Crohn's disease; CRP, C‐reactive protein; IBD, inflammatory bowel disease; MMX, multimatrix; UC, ulcerative colitis.

### 2.1 Pharmacokinetics

Budesonide is a corticosteroid characterised by high first‐pass hepatic metabolism, resulting in low systemic bioavailability and predominantly local anti‐inflammatory effects. It is rapidly distributed, with 85%–90% plasma protein binding and a steady‐state volume of distribution of 3–4 L/kg in both adults and children.^17^, ^18^

Following oral administration, budesonide undergoes extensive first‐pass metabolism via cytochrome P450 3A4 in the liver and intestinal wall, yielding a systemic bioavailability of 10%–20% in CD patients and lower in healthy individuals.^19^ In cirrhosis, bioavailability increases 2.5‐fold due to impaired hepatic metabolism. The drug has a high clearance (~80 L/h) and a plasma half‐life of 2–4.5 h.^20^

Controlled‐release formulations enhance delivery to specific intestinal segments. The pH‐dependent formulation dissolves at pH > 5.5, releasing drug in the ileocecal region. The MMX system allows prolonged release throughout the colon. The controlled ileal‐release version combines time‐ and pH‐dependent mechanisms targeting the terminal ileum and ascending colon.^21^

**Figure 1:**
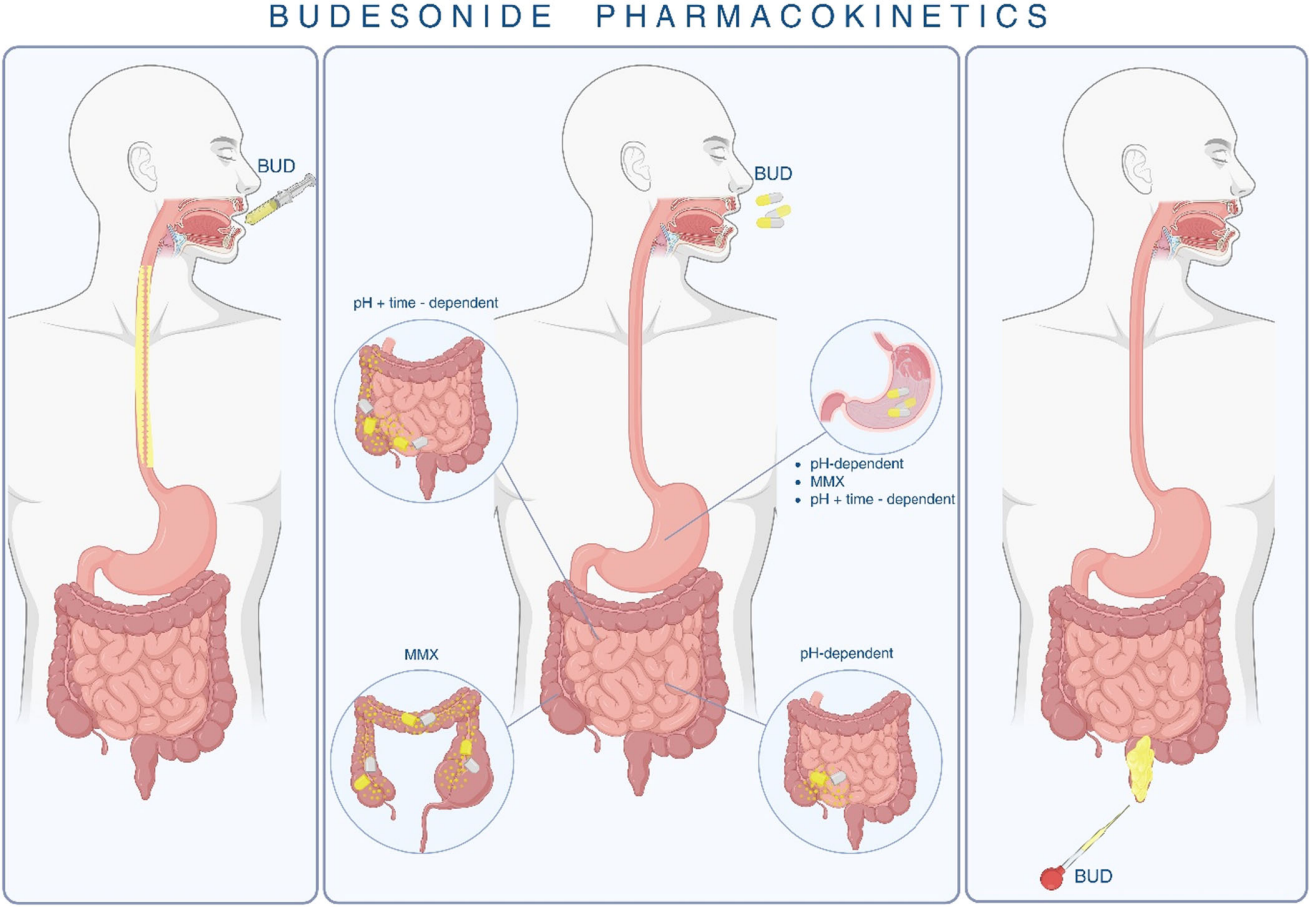
Budesonide Pharmacokinetics: The figure illustrates the targeted delivery and site‐specific absorption of budesonide via various formulations. Oral viscous budesonide is designed for local action in the Oesophagus with minimal systemic absorption. Oral delayed‐release formulations include pH‐dependent, time‐dependent, and MMX systems to target drug release in the small intestine and colon. Rectal formulations, such as enemas and suppositories, facilitate localised delivery to the distal colon and rectum. BUD, budesonide; MMX, multimatrix.

Budesonide is metabolised 2–5 times faster than hydrocortisone and 8–15 times faster than prednisolone, producing the inactive metabolites 6β‐hydroxy‐budesonide and 16α‐hydroxy‐prednisolone, which are primarily excreted renally.^21^ Food delays absorption but does not affect bioavailability.^22^ In children aged 3–6 years, systemic availability from nebulised budesonide is ~6%.^23^ The targeted delivery and site‐specific absorption of budesonide across different formulations are illustrated in Figure 1.

In eosinophilic oesophagitis (EoE), formulations are specifically designed to maximise oesophageal contact. Oral viscous budesonide (OVB), usually compounded with sucralose or xylitol, and orodispersible tablets (OBTs) enhance mucosal delivery and prolong contact time. Importantly, the choice of vehicle significantly influences therapeutic outcomes: OVB achieves longer mucosal contact compared with nebulised budesonid,^24^ while OBTs have demonstrated superior rates of clinico‐histological remission compared with other budesonide formulations in both controlled trials and real‐world practice.^25^, ^26^

### 2.2 Pharmacodynamics

Budesonide exerts its anti‐inflammatory effects through high‐affinity binding to glucocorticoid receptors in the gastrointestinal tract, inhibiting the release of pro‐inflammatory cytokines and mediators such as interleukins (IL), tumour necrosis factor (TNF‐α) and eicosanoids. This suppresses immune cell infiltration and local inflammation, reducing tissue damage.^27^

Compared to conventional corticosteroids, budesonide has a higher local‐to‐systemic activity ratio due to extensive first‐pass metabolism (85%–95%), minimising systemic adverse effects. In inflammatory bowel disease (IBD), pH‐modified release capsules deliver effective local tissue concentrations (15–60 ng/g) in the descending colon, sigmoid and rectum.^21^, ^28^ Its reduced systemic activity leads to less hypothalamic‐pituitary‐adrenal (HPA) axis suppression. Budesonide's systemic effects measured by cortisol and white cell count changes are 2‐ to 4‐fold lower than those of beclomethasone dipropionate after oral administration.^23^, ^29^ This favourable safety profile supports its use in localised ileocaecal CD and EoE, while efficacy in extensive ulcerative colitis (UC) is limited.^30^

In addition to favourable pharmacokinetics, budesonide demonstrates a substantially higher glucocorticoid receptor binding affinity compared to prednisolone, which contributes to its potent local anti‐inflammatory activity.^31^ Clinical studies have further confirmed that budesonide achieves therapeutic efficacy comparable to prednisolone in CD, but with a lower incidence of systemic adverse effects due to its extensive first‐pass metabolism.^32^

**Figure 2:**
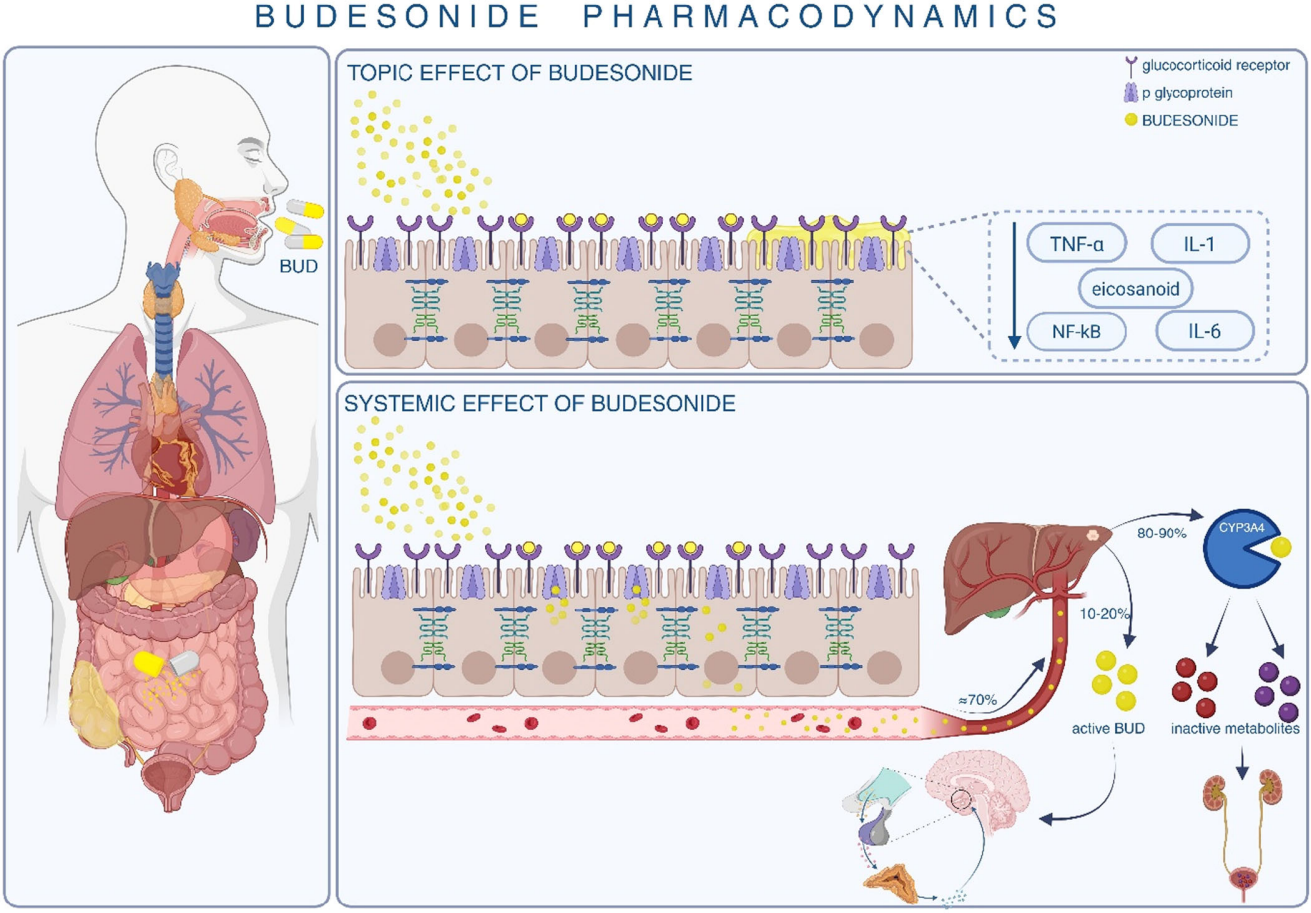
Budesonide pharmacodynamics: Shows the topical and systemic anti‐inflammatory effects of budesonide. It binds to glucocorticoid receptors, suppressing pro‐inflammatory mediators. Systemic absorption undergoes hepatic metabolism via CYP3A4 into inactive metabolites. BUD, budesonide; IL, interleukin; NF‐κB, nuclear factor kappa; TNF‐α, tumour necrosis factor.

In EoE, budesonide acts locally in the oesophageal mucosa by downregulating Th2 cytokines such as IL‐5, IL‐13 and eotaxin‐3. This reduces eosinophilic infiltration and inflammation, contributing to both clinical and histological remission. Studies with oral viscous and orodispersible budesonide confirm reductions in eosinophil counts and cytokine markers, with minimal systemic glucocorticoid activity in most patients.^33^, ^34^, ^35^ The topical and systemic anti‐inflammatory mechanisms of budesonide are depicted in Figure 2.

### 2.3 Indications and efficacy

Budesonide is indicated for mild to moderate CD, UC and EoE in paediatric patients, depending on formulation and regulatory approval. For CD, the ECCO‐ESPGHAN [European Crohn's and Colitis Organisation‐European Society for Pediatric Gastroenterology, Hepatology, and Nutrition] 2020 guideline recommends budesonide as an alternative to exclusive enteral nutrition (EEN) for induction in low‐risk patients with mild ileocaecal disease.^36^ In EoE, recommended paediatric dosing has been suggested as 1 mg twice daily for ages 1–8 years and either 2 mg once daily or 1 mg twice daily for ages 9–17 years. However, it is important to note that no budesonide formulation is currently licensed for EoE in patients under 18 years in Europe, and treatment in this age group frequently relies on master formulas or home‐compounded preparations. In contrast, in the United States, an OVB formulation has been approved for patients aged ≥12 years, with induction therapy typically requiring 2 mg twice daily.^37^ This highlights the discrepancy between guideline‐recommended dosing and the availability of licensed preparations across different regions.

In paediatric UC, oral or rectal budesonide may induce remission in mild left‐sided or distal disease, but is not first‐line therapy. ESPGHAN/ECCO guidelines favour systemic corticosteroids or 5‐aminosalicylic acid (5‐ASA) for moderate to severe disease.^38^ Budesonide may be used when 5‐ASA is not tolerated or systemic corticosteroids are contraindicated. Its maintenance efficacy remains unproven, and rectal budesonide is less effective than rectal 5‐ASA.^39^

Although budesonide is first‐line for microscopic colitis in adults, its use in paediatric microscopic colitis and autoimmune hepatitis is off‐label and supported by limited evidence.^40^, ^41^

## 3 EOSINOPHILIC ESOPHAGITIS

Several studies have evaluated the efficacy and safety of OVB in paediatric EoE. In a retrospective study by Aceves et al., 20 children (mean age 5.5 years) showed significant improvement in eosinophil counts (from 87 to 7 eos/hpf), symptom scores (from 4.4 to 0.8), and endoscopic findings. No significant adverse events were reported, and cortisol levels remained within normal range.^4^

Dohil et al. conducted a randomised, placebo‐controlled trial in 24 children, where 15 received OVB and 9 placebo for 3 months. Budesonide significantly reduced eosinophil counts (from 66.7 to 4.8 eos/hpf) and improved symptom and endoscopic scores compared to placebo.^5^

Oliva et al. and Warzecha et al. confirmed histological remission following 8–12 weeks of OVB therapy in small pilot cohorts. In a follow‐up study, Oliva et al. enroled 20 patients (median age 10 years) in a 12‐week induction phase followed by a reduced‐dose 12‐week maintenance phase. Histological remission (peak eosinophils <6 eos/hpf) was achieved in 90% after induction, with 85% maintaining remission at 24 weeks, but only 45% by week 36, indicating risk of relapse posttreatment.^6^, ^9^, ^10^ Gupta et al. evaluated dose‐ranging oral budesonide suspension (0.35–2.8 mg twice daily) versus placebo in 71 children aged 2–18 years. Significant histologic response (≤16 eos/hpf) was observed in medium‐ and high‐dose groups, though symptom scores did not differ significantly. No major adverse events were reported.^7^ Tambucci et al. assessed 12 weeks of OVB in children with EoE and oesophageal atresia, showing histological remission in 87.5% and improvement in clinical and endoscopic features without reported safety concerns.^8^

In 2024, the US FDA approved EOHILIA, a ready‐to‐use oral budesonide suspension, for short‐term treatment of EoE in patients aged 11 years and older, based on phase 3 data demonstrating clinical and histological efficacy.^42^ ESPGHAN/NASPGHAN [North American Society for Pediatric Gastroenterology, Hepatology, and Nutrition] guidelines also conditionally recommend topical corticosteroids, including budesonide, for selected cases of non‐EoE eosinophilic gastrointestinal disorders (EGIDs).^43^ The recent approval of dupilumab for children aged ≥1 year with EoE has significantly shifted the therapeutic paradigm.^38^, ^44^ Unlike budesonide, dupilumab targets the underlying Th2‐driven inflammatory pathway and has demonstrated durable improvements in histology, symptoms and quality of life. Importantly, dupilumab avoids the risk of HPA axis suppression associated with long‐term corticosteroid use.^44^ However, considerations such as high cost, need for subcutaneous injection, and limited global availability currently restrict its universal adoption. In clinical practice, budesonide remains an accessible and effective first‐line therapy for induction,^6^, ^9^, ^10^ whereas dupilumab may increasingly be considered for patients with relapsing disease, steroid‐related side effects, or significant comorbid atopy.

While generally safe, rare cases of oesophageal or oral candidiasis have been reported.^4^, ^5^ Although multiple studies demonstrate the efficacy of OVB in inducing histological remission, the majority are small retrospective or pilot trials with limited follow‐up (typically ≤12 weeks). Few RCTs exist, and many studies rely on surrogate endpoints such as eosinophil counts without long‐term clinical outcomes. This limits the generalisability of findings and underscores the need for larger, multicentred RCTs with extended follow‐up.

It is important to emphasise that studies evaluating budesonide in paediatric EoE have used a variety of formulations, including oral viscous suspensions, nebulised solutions, OBTs and other compounded preparations. These formulations differ not only in dose but also in vehicle composition, with many relying on master formulas or home‐prepared suspensions. Such heterogeneity may contribute to variability in clinical and histological outcomes reported across studies.^45^ This underscores the need for standardised, commercially available paediatric formulations to ensure reproducibility and optimise therapeutic outcomes.

## 4 PAEDIATRIC IBD

Budesonide presents a lower risk of side effects and adrenal suppression (AS) compared to conventional corticosteroids, making it a preferable option in paediatric CD, especially considering the growth and bone health concerns associated with prednisone.^13^

Levine et al. reported clinical response rates of 51.4% and remission in 42.9% with 9 mg/day of budesonide, compared to 74.3% response and 65.7% remission with 12 mg/day. The higher dose also showed greater reductions in C‐reactive protein without increased adverse effects or cortisol suppression.^11^

Cohen et al. conducted a multicentre trial evaluating enteric‐coated budesonide in children with mild to moderate CD. Patients received either 9 or 6 mg daily during an 8‐week induction phase, followed by 6 mg daily for 12 weeks of maintenance. Budesonide was well tolerated, though 31.1% (32/103) had subnormal morning cortisol after 8 weeks, indicating some risk of AS. Remission (PCDAI < 10) was achieved in 58.1% during induction, but was not sustained during maintenance.^12^

Growth retardation and reduced final height are important concerns with corticosteroid use in children. Studies suggest that budesonide induces similar remission rates to prednisone but with fewer systemic side effects and less HPA axis suppression.^46^

Data from the Paediatric IBD Collaborative Research Group Registry showed that budesonide was used in 13% of newly diagnosed CD patients, mostly within 30 days of diagnosis.^10^ It was used as monotherapy in only 5% of cases, with most patients receiving it as part of combination therapy. While budesonide remains a useful option for mild, low‐risk ileocecal CD,^46^ biologics such as infliximab are increasingly recommended as first‐line induction and maintenance therapy in children with high‐risk features.^36^ Compared with budesonide, infliximab achieves higher rates of sustained remission and mucosal healing, but carries greater risks of immunosuppression and requires intravenous administration. Thus, budesonide should be viewed as an induction option for carefully selected, low‐risk patients, whereas biologics are prioritised in more aggressive disease phenotypes.

In addition to individual randomised trials, systematic reviews and meta‐analyses have evaluated budesonide for induction and maintenance of remission in CD,^12^, ^46^ including subanalyses in paediatric populations. While these provide pooled efficacy and safety estimates, the present review focuses on summarising data from individual RCTs and observational studies to allow a more detailed consideration of study designs and outcomes.

Although budesonide shows favourable safety compared to systemic corticosteroids, most available trials in paediatric CD are limited by modest sample sizes and heterogeneity in endpoints. Maintenance efficacy is less consistently demonstrated, reflecting both study design variability and the challenges of long‐term adherence in children.

## 5 UC

Data on budesonide use in paediatric UC are limited. In general, budesonide is less effective than prednisolone for inducing remission in mild to moderate UC, though it is superior to placebo and mesalamine in some studies. According to ESPGHAN guidelines, steroid enemas are less effective than 5‐ASA enemas (1 g/day) in paediatric distal colitis.^39^ Although rectal corticosteroid preparations (e.g., budesonide or beclomethasone foams/enemas) may offer benefit, most evidence comes from adult trials, and paediatric data remain sparse.^27^

Adamczuk et al. evaluated nine paediatric UC patients (mean age 7.5 years) treated with budesonide MMX for 8 weeks. Clinical remission was achieved in 77.7% by Week 4 and sustained through Week 8, indicating good efficacy and tolerability.^14^ In a larger cohort, Meglicka et al. studied 31 paediatric UC patients (mean age 13.2 years) treated with budesonide MMX for 2 months. Clinical remission (PUCAI < 10) occurred in 55%, endoscopic improvement in 73%, and endoscopic remission in 40%. However, patients treated beyond 10 months experienced adverse events, including weight gain and Cushingoid features, highlighting the risks of prolonged use.^15^

Karolewska‐Bochenek et al. followed 16 paediatric UC patients (median age 14.5 years) with a median disease duration of 18 months. Only one patient achieved clinical remission after 8 weeks, suggesting limited efficacy, although the treatment was well tolerated.^16^

Evidence in paediatric UC remains sparse and largely extrapolated from adult trials. The few paediatric studies conducted to date are underpowered and vary in treatment duration, making it difficult to draw firm conclusions on long‐term efficacy and safety.

## 6 MICROSCOPIC COLITIS

Over the past two decades, microscopic colitis (MC) has emerged as a common cause of chronic, nonbloody diarrhoea in adults, with incidence rates approaching those of UC and CD. Budesonide is the most effective treatment for both collagenous colitis (CC) and lymphocytic colitis (LC), with multiple RCTs supporting its use for induction and maintenance of remission.

In adults, remission rates of 85% and 77% have been reported with budesonide, significantly outperforming placebo.^47^ A meta‐analysis confirmed a pooled remission rate of 81% for induction therapy, and extended treatment reduced recurrence, with one study showing 61.4% of patients in remission at 12 months versus 16.7% with placebo.^48^ Even low‐dose maintenance (e.g., 4.5 mg/day) was effective and well tolerated.^49^

Adverse events were generally mild headache, nausea, acne and corticosteroid‐related effects such as moon face and occurred at similar rates to placebo. Enteric‐coated formulations had minimal impact on blood cortisol levels. Despite its efficacy, relapse upon discontinuation is common, supporting the need for tailored maintenance strategies.

European consensus guidelines recommend budesonide as the first‐line therapy for both induction and maintenance of remission in MC, owing to its local anti‐inflammatory action and limited systemic absorption.^50^

However, its use in paediatric MC remains off‐label, with little available data. By contrast, several small studies in paediatric IBD populations (CD and UC) suggest budesonide offers clinical benefit with a favourable safety profile compared to systemic corticosteroids. Larger multicentre trials are needed to clarify its role in paediatric MC and broader IBD care.

Although multiple RCTs confirm efficacy in adults, the lack of paediatric data and reliance on small, retrospective case series limits applicability in younger populations. An overview of the dosing regimens, typical treatment durations and supporting levels of evidence across the four major gastrointestinal indications is presented in Table S1, highlighting important differences between disease contexts.

## 7 SAFETY AND ADRENAL AXIS SUPPRESSION IN BUDESONIDE THERAPY

Budesonide, a potent glucocorticoid with high first‐pass metabolism, is favoured in paediatric IBD for its strong local anti‐inflammatory effects and reduced systemic exposure. However, systemic absorption can still occur, particularly when combined with CYP3A4 inhibitors like itraconazole. A study by Anjum and Jakoby found HPA axis suppression in 44% of patients co‐treated with budesonide and itraconazole, underscoring the need for close monitoring.^51^

AS is a known but often underrecognised complication of long‐term corticosteroid therapy. Symptoms may be nonspecific until physiological stress precipitates an adrenal crisis marked by hypotension, hypoglycaemia, seizures, or coma. Inhaled budesonide doses >1–1.5 mg/day have been linked to biochemical AS in up to 46% of children with asthma. Despite similar concerns, formal screening guidelines do not exist for OVB.^52^

Harel et al. demonstrated that 43% of 19 paediatric EoE patients on OVB for ≥3 months showed abnormal cortisol responses to ACTH stimulation, regardless of dose, treatment duration, or concomitant inhaled steroid use. Notably, two patients remained suppressed even 2–6 months after discontinuing therapy. These findings suggest a prolonged impact on adrenal function and highlight the need for routine screening in long‐term OVB users, particularly before surgery or during severe illness.^52^

AS is a recognised concern with long‐term budesonide use, although its clinical relevance varies depending on the formulation, dose, duration and patient monitoring. Importantly, reported prevalence rates depend on whether appropriate suppression tests are employed. In EoE, a meta‐analysis by Philpott et al. demonstrated that topical corticosteroid therapy, including budesonide, was associated with an increased risk of AS, though often subclinical and reversible. These findings underscore the need for careful monitoring when budesonide is used chronically in paediatric populations.^53^, ^54^

The ACTH stimulation test remains the most sensitive diagnostic tool, while morning cortisol levels have limited sensitivity (~60%). Preventive strategies should include dose tapering, perioperative stress dosing with hydrocortisone (30–50 mg/m²/day) and patient/caregiver education. Systemic corticosteroid exposure from other sources (e.g., inhaled, topical, and nasal) and altered CYP3A metabolism in EoE may also contribute to interpatient variability and risk.^53^, ^54^ Given the absence of specific guidelines, further research is needed to establish safe monitoring protocols and compare OVB with alternatives like fluticasone.

When weighed against newer biologics, budesonide offers advantages of oral/topical administration and relative affordability, but lacks the long‐term disease‐modifying potential of agents such as infliximab in CD^36^ or dupilumab in EoE.^38^, ^39^, ^44^ Thus, its role may best be defined as a short‐term induction therapy in mild to moderate disease, with biologics reserved for high‐risk or refractory cases.

## 8 CONCLUSION

Despite its favourable safety profile compared to systemic corticosteroids, budesonide is not without risk. Long‐term use, particularly in children, can lead to adverse events such as HPA axis suppression, growth retardation and, rarely, metabolic complications. Therefore, its use should be closely monitored, with attention to cumulative steroid exposure and the potential need for adrenal function assessment in long‐term users.

Multiple studies support the efficacy of OVB in paediatric EoE, showing both histological and clinical remission. Despite small sample sizes, findings consistently report reductions in eosinophil counts alongside improvements in symptoms and endoscopic appearance, underscoring its safety and effectiveness. However, variability in maintaining long‐term remission remains a concern.

Emerging off‐label use of crushed or opened budesonide capsules for EGIDs beyond the oesophagus offers promising outcomes. Preliminary evidence suggests benefits in reducing gastric eosinophilic inflammation while limiting systemic steroid exposure and easing dietary restrictions, although further research is needed to confirm long‐term safety and efficacy.

Overall, while budesonide demonstrates efficacy across several gastrointestinal disorders, the current evidence base is heterogeneous. Many paediatric studies are constrained by small sample sizes, short follow‐up, or nonrandomised designs, necessitating cautious interpretation. Future large‐scale, multicentre randomised trials will be essential to establish robust long‐term efficacy and safety profiles.

## CONFLICT OF INTEREST STATEMENT

The authors declare no conflicts of interest.

## Supporting information


**Supplemental Table S1:** Overview of oral budesonide dosing regimens across gastrointestinal disorders.

## References

[1] Löfberg R , Danielsson A , Salde L . Oral budesonide in active Crohn's disease. Aliment Pharmacol Ther. 1993;7(6):611‐616. 8161666 10.1111/j.1365-2036.1993.tb00141.x

[2] Kwapisz L , Jairath V , Khanna R , Feagan B . Pharmacokinetic drug evaluation of budesonide in the treatment of Crohn's disease. Expert Opin Drug Metab Toxicol. 2017;13(7):793‐801. 28612627 10.1080/17425255.2017.1340454

[3] Silverman J , Otley A . Budesonide in the treatment of inflammatory bowel disease. Expert Rev Clin Immunol. 2011;7(4):419‐428. 21790284 10.1586/eci.11.34

[4] Aceves SS , Bastian JF , Newbury RO , Dohil R . Oral viscous budesonide: a potential new therapy for eosinophilic esophagitis in children. Am J Gastroenterol. 2007;102(10):2271‐2279. 17581266 10.1111/j.1572-0241.2007.01379.x

[5] Dohil R , Newbury R , Fox L , Bastian J , Aceves S . Oral viscous budesonide is effective in children with eosinophilic esophagitis in a randomized, placebo‐controlled trial. Gastroenterology. 2010;139(2):418‐429.e1. 20457157 10.1053/j.gastro.2010.05.001

[6] Oliva S , Rossetti D , Papoff P , et al. A 12‐week maintenance therapy with a new prepared viscous budesonide in pediatric eosinophilic esophagitis. Dig Dis Sci. 2019;64:1571‐1578. 30659470 10.1007/s10620-018-5449-x PMC6522447

[7] Gupta SK , Vitanza JM , Collins MH . Efficacy and safety of oral budesonide suspension in pediatric patients with eosinophilic esophagitis. Clin Gastroenterol Hepatol. 2015;13(1):66‐76.e3. 24907502 10.1016/j.cgh.2014.05.021

[8] Tambucci R , Roversi M , Rea F , et al. Oral viscous budesonide in children with eosinophilic esophagitis after repaired esophageal atresia: a clinical trial. J Pediatr Gastroenterol Nutr. 2023;77(2):249‐255. 37195886 10.1097/MPG.0000000000003830

[9] Oliva S , Rossetti D , Papoff P , et al. A new formulation of oral viscous budesonide in treating paediatric eosinophilic oesophagitis: a pilot study. J Pediatr Gastroenterol Nutr. 2017;64(2):218‐224. 27253660 10.1097/MPG.0000000000001281

[10] Warzecha J , Dziekiewicz M , Bieńkowska‐Tokarczyk A , Małecki M , Banaszkiewicz A . A new viscous budesonide formulation for the treatment of eosinophilic esophagitis in children: a preliminary experience and review of the literature. J Clin Med. 2022;11(22):6730. 36431208 10.3390/jcm11226730 PMC9694526

[11] Levine A , Kori M , Dinari G , et al. Comparison of two dosing methods for induction of response and remission with oral budesonide in active pediatric Crohnʼs disease: arandomized placebo‐controlled trial. Inflamm Bowel Dis. 2009;15(7):1055‐1061. 19229988 10.1002/ibd.20881

[12] Cohen SA , Aloi M , Arumugam R , et al. Enteric‐coated budesonide for the induction and maintenance of remission of Crohn's disease in children. Curr Med Res Opin. 2017;33(7):1261‐1268. 28420280 10.1080/03007995.2017.1313213

[13] Otley A , LeLeiko N , Langton C , et al. Budesonide use in pediatric Crohn disease. J Pediatr Gastroenterol Nutr. 2012;55(2):200‐204. 22258289 10.1097/MPG.0b013e31824a09c2

[14] Adamczuk A , Dądalski M , Kierkuś J . Budesonide MMX in pediatric ulcerative colitis. Postępy Nauk Medycznych. 2016;29(4):231‐233.

[15] Meglicka M , Dadalski M , Adamczuk A , Kierkus J . P359 budesonide MMX in paediatric ulcerative colitis. J Crohn's Col. 2019;13(suppl_1):S283‐S284.

[16] Karolewska‐Bochenek K , Dziekiewicz M , Banaszkiewicz A . Budesonide MMX in paediatric patients with ulcerative colitis. J Crohn's Colitis. 2017;11(11):1402. 28505293 10.1093/ecco-jcc/jjx069

[17] Edsbäcker S , Andersson T . Pharmacokinetics of budesonide (Entocort™ EC) capsules for Crohn's disease. Clin Pharmacokinet. 2004;43:803‐821. 15355126 10.2165/00003088-200443120-00003

[18] Gupta SK , Hill M , Vitanza JM , et al. Pharmacokinetics of budesonide oral suspension in children and adolescents with eosinophilic esophagitis. J Pediatr Gastroenterol Nutr. 2022;75(2):186‐191. 35666852 10.1097/MPG.0000000000003482 PMC9278710

[19] Williams DM . Clinical pharmacology of corticosteroids. Respir Care. 2018;63(6):655‐670. 29794202 10.4187/respcare.06314

[20] Liu D , Bonwick WMW , Sumithran P , Grace JA , Sinclair M . Budesonide in liver immunology: a therapeutic opportunity in liver transplantation. Curr Transplant Rep. 2024;11(4):197‐206.

[21] Quetglas EG , Armuzzi A , Wigge S , et al. Review article: the pharmacokinetics and pharmacodynamics of drugs used in inflammatory bowel disease treatment. Eur J Clin Pharmacol. 2015;71:773‐799. 26008212 10.1007/s00228-015-1862-7

[22] Song IH , Finkelman RD , Lan L . A pharmacokinetic bridging study to compare systemic exposure to budesonide between budesonide oral suspension and ENTOCORT EC in healthy individuals. Drugs R&D. 2020;20:359‐367. 10.1007/s40268-020-00324-1 PMC7691405 33057953

[23] Szefler SJ . Pharmacodynamics and pharmacokinetics of budesonide: a new nebulized corticosteroid. J Allergy Clin Immunol. 1999;104(4):S175‐S183. 10.1016/s0091-6749(99)70059-x 10518844

[24] Dellon ES , Sheikh A , Speck O , et al. Viscous topical is more effective than nebulized steroid therapy for patients with eosinophilic esophagitis. Gastroenterology. 2012;143(2):321‐324.e1. 22561055 10.1053/j.gastro.2012.04.049 PMC3404241

[25] Miehlke S , Lucendo AJ , Straumann A , Jan Bredenoord A , Attwood S . Orodispersible budesonide tablets for the treatment of eosinophilic esophagitis: a review of the latest evidence. Therap Adv Gastroenterol. 2020;13:1756284820927282. 10.1177/1756284820927282 PMC7288799 32565912

[26] Laserna‐Mendieta EJ , Navarro P , Casabona‐Francés S , et al. Swallowed topical corticosteroids for eosinophilic esophagitis: utilization and real‐world efficacy from the EoE CONNECT registry. United European Gastroenterol J. 2024;12(5):585‐595. 10.1002/ueg2.12533 PMC11176909 38284792

[27] Iborra M , Álvarez‐Sotomayor D , Nos P . Long‐term safety and efficacy of budesonide in the treatment of ulcerative colitis. Clin Exp Gastroenterol. 2014;7:39‐46. 24523594 10.2147/CEG.S34715 PMC3921089

[28] Edsbäcker S , Bengtsson B , Larsson P , et al. A pharmacoscintigraphic evaluation of oral budesonide given as controlled‐release (Entocort) capsules. Aliment Pharmacol Ther. 2003;17(4):525‐536. 12622761 10.1046/j.1365-2036.2003.01426.x

[29] Stark JG , Werner S , Homrighausen S , et al. Pharmacokinetic/pharmacodynamic modeling of total lymphocytes and selected subtypes after oral budesonide. J Pharmacokinet Pharmacodyn. 2006;33:441‐459. 16633890 10.1007/s10928-006-9013-5

[30] Dignass A , Van Assche G , Lindsay JO , et al. The second European evidence‐based consensus on the diagnosis and management of Crohn's disease: current management. J Crohn's Col. 2010;4(1):28‐62. 10.1016/j.crohns.2009.12.002 21122489

[31] Johansson SÅ , Andersson KE , Brattsand R , Gruvstad E , Hedner P . Topical and systemic glucocorticoid potencies of budesonide and beclomethasone dipropionate in man. Eur J Clin Pharmacol. 1982;22(6):523‐529. 7128664 10.1007/BF00609625

[32] Greenberg GR , Feagan BG , Martin F , et al. Oral budesonide for active Crohn's disease. N Engl J Med. 1994;331(13):836‐841. 8078529 10.1056/NEJM199409293311303

[33] Dellon ES , Woosley JT , Arrington A , et al. Efficacy of budesonide vs fluticasone for initial treatment of eosinophilic esophagitis in a randomized controlled trial. Gastroenterology. 2019;157(1):65‐73.e5. 30872104 10.1053/j.gastro.2019.03.014 PMC6581596

[34] Hirano I , Collins MH , Katzka DA , et al. Budesonide oral suspension improves outcomes in patients with eosinophilic esophagitis: results from a phase 3 trial. Clin Gastroenterol Hepatol. 2022;20(3):525‐534.e10. 33887475 10.1016/j.cgh.2021.04.022

[35] Straumann A , Lucendo AJ , Miehlke S , et al. Budesonide orodispersible tablets maintain remission in a randomized, placebo‐controlled trial of patients with eosinophilic esophagitis. Gastroenterology. 2020;159(5):1672‐1685.e5. 32721437 10.1053/j.gastro.2020.07.039

[36] Van Rheenen PF , Aloi M , Assa A , et al. The medical management of paediatric Crohn's disease: an ECCO‐ESPGHAN guideline update. J Crohn's Col. 2021;15(2):171‐194. 10.1093/ecco-jcc/jjaa161 33026087

[37] Dellon ES , Katzka DA , Mukkada VA , et al. Long‐term safety and efficacy of budesonide oral suspension for eosinophilic esophagitis: a 4‐year, phase 3, open‐label study. Clin Gastroenterol Hepatol. 2025;23(12):2155‐2166. 10.1016/j.cgh.2024.12.024 39954913

[38] Amil‐Dias J , Oliva S , Papadopoulou A , et al. Diagnosis and management of eosinophilic esophagitis in children: an update from the European Society for Paediatric Gastroenterology, Hepatology and Nutrition (ESPGHAN). J Pediatr Gastroenterol Nutr. 2024;79(2):394‐437. 38923067 10.1002/jpn3.12188

[39] Turner D , Ruemmele FM , Orlanski‐Meyer E , et al. Management of paediatric ulcerative colitis, part 1: ambulatory care—an evidence‐based guideline from European Crohn's and Colitis Organization and European Society of Paediatric Gastroenterology, Hepatology and Nutrition. J Pediatr Gastroenterol Nutr. 2018;67(2):257‐291. 30044357 10.1097/MPG.0000000000002035

[40] Abdalla MI , Herfarth H . Budesonide for the treatment of ulcerative colitis. Expert Opin Pharmacother. 2016;17(11):1549‐1559. 27157244 10.1080/14656566.2016.1183648 PMC4989907

[41] Remien KA , Mancuso M , Watson K . Pediatric collagenous gastroenteritis and colitis presenting as protein‐losing enteropathy. ACG Case Rep J. 2023;10(4):e01028. 37057196 10.14309/crj.0000000000001028 PMC10090787

[42] Budesonide oral suspension (Eohilia) for eosinophilic esophagitis. Med Lett Drugs Ther. 2024;66(1704):93‐95. 10.58347/tml.2024.1704c 38905533

[43] Papadopoulou A , Amil‐Dias J , Auth MKH , et al. Joint ESPGHAN/NASPGHAN guidelines on childhood eosinophilic gastrointestinal disorders beyond eosinophilic esophagitis. J Pediatr Gastroenterol Nutr. 2024;78(1):122‐152. 38291684 10.1097/MPG.0000000000003877

[44] Harel S , Hursh BE , Chan ES , Avinashi V , Panagiotopoulos C . Adrenal suppression in children treated with oral viscous budesonide for eosinophilic esophagitis. J Pediatr Gastroenterol Nutr. 2015;61(2):190‐193. 25950088 10.1097/MPG.0000000000000848

[45] Joshi S , Rubenstein JH , Dellon ES , Worthing N , Stefanadis Z , Chang JW . Variability in practices of compounding budesonide for eosinophilic esophagitis. Am J Gastroenterol. 2021;116(6):1336‐1338. 33538420 10.14309/ajg.0000000000001170 PMC9161310

[46] Rezaie A , Kuenzig ME , Benchimol EI , et al. Budesonide for induction of remission in Crohn's disease. Cochrane Database Syst Rev. 2015;2015(6):CD000296. 26039678 10.1002/14651858.CD000296.pub4 PMC10613338

[47] Miehlke S , Madisch A , Kupcinskas L , et al. Budesonide is more effective than mesalamine or placebo in short‐term treatment of collagenous colitis. Gastroenterology. 2014;146(5):1222‐1230.e2. 24440672 10.1053/j.gastro.2014.01.019

[48] Sebastian S , Wilhelm A , Jessica L , Myers S , Veysey M . Budesonide treatment for microscopic colitis: systematic review and meta‐analysis. Eur J Gastroenterol Hepatol. 2019;31(8):919‐927. 31211724 10.1097/MEG.0000000000001456

[49] Münch A , Bohr J , Miehlke S , et al. Low‐dose budesonide for maintenance of clinical remission in collagenous colitis: a randomised, placebo‐controlled, 12‐month trial. Gut. 2016;65(1):47‐56. 25425655 10.1136/gutjnl-2014-308363 PMC4717436

[50] Miehlke S , Guagnozzi D , Zabana Y , et al. European guidelines on microscopic colitis: United European Gastroenterology and European Microscopic Colitis Group statements and recommendations. United European Gastroenterol J. 2021;9(1):13‐37. 10.1177/2050640620951905 PMC8259259 33619914

[51] Anjum F , Jakoby MG . 7432 secondary adrenal insufficiency caused by concurrent treatment with budesonide and itraconazole. J Endocr Soc. 2024;8(suppl_1):bvae163‐bvae287.

[52] Harel S , Hursh BE , Chan ES , Avinashi V , Panagiotopoulos C . Adrenal suppression in children treated with oral viscous budesonide for eosinophilic esophagitis. J Pediatr Gastroenterol Nutr. 2015;61(2):190‐193. 25950088 10.1097/MPG.0000000000000848

[53] Philpott H , Dougherty MK , Reed CC , et al. Systematic review: adrenal insufficiency secondary to swallowed topical corticosteroids in eosinophilic oesophagitis. Aliment Pharmacol Ther. 2018;47(8):1071‐1078. 29508432 10.1111/apt.14573 PMC5867261

[54] Kennedy K , Muir AB , Grossman A , et al. Modified oral enteric‐coated budesonide regimens to treat pediatric eosinophilic gastroenteritis, a single center experience. J Allergy Clin Immunol Pract. 2019;7(6):2059‐2061. 30763733 10.1016/j.jaip.2019.01.053 PMC7064262

