# Supplementary material for: Drugs in focus: Budesonide and its role in paediatric gastrointestinal disorders
Source: J Pediatr Gastroenterol Nutr. 2025 Oct 27;82(1):60–9. doi: 10.1002/jpn3.70245 (PMC12780467; doi:10.1002/jpn3.70245)
Supplement: Supplementary file 1 — Supplemental Table S1: Overview of oral budesonide dosing regimens across gastrointestinal disorders. [file JPN3-82-60-s001.docx]

**Supplemental Table S1:** Overview of oral budesonide dosing regimens across gastrointestinal disorders.

| Disease | Typical Dosing Regimen | Usual Duration | Level of Evidence |
| --- | --- | --- | --- |
| Crohn’s disease (paediatric) | Oral controlled-release 9 mg/day (induction); up to 12 mg/day in some trials; taper to 6 mg/day for maintenance | 8 weeks induction; up to 12 weeks maintenance | **Moderate** (several RCTs, guideline-supported for mild ileocecal CD) |
| Ulcerative colitis (paediatric) | Budesonide-MMX 9 mg/day; rectal foam/enema 2 mg/day | 8 weeks induction; limited/unsafe beyond 10 months | **Low–Moderate** (few paediatric studies; mostly adult data extrapolated) |
| Eosinophilic esophagitis (paediatric) | Oral viscous budesonide (OVB) 1 mg BID (ages 1–8); 2 mg/day or 1 mg BID (ages 9–17); FDA-approved oral suspension 2 mg BID (≥11 years) | 8–12 weeks induction; relapse common post-discontinuation | **Moderate** (small RCTs, multiple observational studies, FDA approval ≥11y) |
| Microscopic colitis (paediatric, off-label) | Oral controlled-release 9 mg/day induction; 6 mg/day or 4.5 mg/day for maintenance (adult data) | 6–8 weeks induction; maintenance up to 12 months in adults | **High (adults)**; **Very Low (paediatrics)** |
